# Adaptive Collaborative Gaussian Mixture Probability Hypothesis Density Filter for Multi-Target Tracking

**DOI:** 10.3390/s16101666

**Published:** 2016-10-11

**Authors:** Feng Yang, Yongqi Wang, Hao Chen, Pengyan Zhang, Yan Liang

**Affiliations:** 1 School of Automation, Northwestern Polytechnical University, Xi’an 710072, China; yangfeng@nwpu.edu.cn (F.Y.); chenhao0727@mail.nwpu.edu.cn (H.C.); yybingxueer@163.com (P.Z.); 2 Key Laboratory of Information Fusion Technology, Ministry of China, Xi’an 710072, China; 3 Southwest China Research Institute of Electronic Equipment (SWIEE), Chengdu 610036, China; wangyongqi12@163.com

**Keywords:** multi-target tracking, multi-target state and track extraction, GMPHD filter

## Abstract

In this paper, an adaptive collaborative Gaussian Mixture Probability Hypothesis Density (ACo-GMPHD) filter is proposed for multi-target tracking with automatic track extraction. Based on the evolutionary difference between the persistent targets and the birth targets, the measurements are adaptively partitioned into two parts, persistent and birth measurement sets, for updating the persistent and birth target Probability Hypothesis Density, respectively. Furthermore, the collaboration mechanism of multiple probability hypothesis density (PHDs) is established, where tracks can be automatically extracted. Simulation results reveal that the proposed filter yields considerable computational savings in processing requirements and significant improvement in tracking accuracy.

## 1. Introduction

In multi-target tracking (MTT) in clutters, the correspondence between the targets and the measurements is unknown, while the target number is unknown and even time-varying. The objective of MTT is to recursively estimate the target number and target states from a sequence of noisy and cluttered measurement sets [1,2].

One common approach for multi-target tracking is the combination of state estimation and data association, along with track initialization and termination [3]. In fact, data association and state estimation are coupled issues, i.e., the association risk triggers the measurement misuse and the estimation error increases the association risk. In other words, their direct combination above is in principle not suitable to the high uncertainty case; for example, dense targets/dense clutter [4].

Another approach is to apply random finite sets (RFSs) to represent the collection of individual targets and measurements, and hence recast the MTT problem as the Bayesian estimation problem based on finite set statistics so as to avoid data association risk [4]. However, the propagation of the multi-target posterior probability density function (PDF) is computationally intensive, which stems from the high-dimension integrations in multi-target state space. Mahler [5] proposed the first-order moment called the probability hypothesis density (PHD) of the PDF of the random set of state vectors. Vo and Ma [6] proved that the PHD surface is a Gaussian mixture (GM) in both the linear and Gaussian cases. Clark and Vo [7] analyzed the convergence property of the Gaussian Mixture Probability Hypothesis Density (GMPHD) filter. Up to now, PHD-related applications have been extended to many fields including visual target tracking [8], maneuvering target tracking [9,10], ground target tracking [11], extended target tracking [12,13], and sensor management [14].

However, there are still two important but open issues about PHD:

The first is that the computational burden is still too intensive in many actual applications, especially in the case of dense clutters and intensive targets. One possible solution is to discern the measurement originality based on tracking gating [15,16]. Nevertheless, such a decision still faces mistake risks.

The second issue is that additional track extraction is needed because the standard PHD only outputs the track points without the corresponding track identities. But the presented track extraction algorithm [17,18] is too complex to implement.

In this paper, we present an adaptive collaborative GMPHD (ACo-GMPHD) filter with the capability for automatic track extraction for fast multi-target tracking in dense clutter. In the ACo-GMPHD, the persistent and birth target PHDs are updated respectively based on the corresponding measurement subsets, instead of based on all measurements, so that the computational complexity is expected to be reduced. Meanwhile, the collaboration mechanism among these PHDs regarding measurement utilization is adaptively established to avoid decision risks triggered by measurement partitions. In addition, permanent tracks and temporary tracks can be automatically extracted from the persistent and birth target PHDs. Simulations show that the proposed ACo-GMPHD greatly reduces the computational cost and significantly improves the track extraction, compared with the well-known GMPHD filter.

The remainder of this work is organized as follows. Section 2 presents the MTT problem and briefly introduces the standard PHD filter. The ACo-GMPHD is proposed in Section 3, and compared with the GMPHD filter via simulation in Section 4. Finally, Section 5 concludes this paper.

## 2. Problem Formulation

### 2.1. Additional Gaussian Noise Model

In an MTT scenario, targets appear and disappear randomly. A new target appears in the surveillance region either by spontaneous birth or by spawning from an existing target. The number of the newly-born targets is assumed to be Poisson-distribution. A target may disappear at the next instant. Here, $p_{S,k}(x_{k-1})$ represents the probability that a target corresponding to the state $x_{k-1}$ at k−1 survives up to k. For a target in the surveillance region, its movement is depicted by an additional Gaussian noise model:

(1) $$p_{k|k-1}\left(x|\zeta \right)=N\left(x;f(\zeta ),Q_{k-1}\right)$$

define N(⋅;m,P) is a Gaussian density function with mean m and covariance P, so here, m=f(ξ), $P=Q_{k-1}$; One corresponding measurement will be obtained according to the following additional Gaussian noise model:

(2) $$p_{k}(z|x)=N\left(z;h(x),R_{k}\right)$$

where f is the state mapping for k−1 to k; $Q_{k-1}$ is the covariance of process noise; h is the mapping from the state to the measurement; and $R_{k}$ is the covariance of measurement noise.

Denote $x_{k,i}$ as the i-th target state, $z_{k,j}$ as the *j*-th measurement, and $N_{k}$ and $M_{k}$ as the target number and measurement number, respectively. Now the multi-target states and observation sensor measurements are represented by the random finite sets (RFS) $X_{k}=\left\{x_{k,1},\ldots ,x_{k,N_{k}}\right\}\in ℱ\left(X\right)$ and $Z_{k}=\left\{z_{k,1},\ldots ,z_{k,M_{k}}\right\}\in ℱ\left(Z\right)$, respectively. Here, ℱ(X) and ℱ(Z) are the space of all finite subsets of state space X and measurement space Z, respectively. The RFS of the target states is as stated in [19]:

(3) $$X_{k}=\left[\underset{\zeta \in X_{k-1}}{⋃}S_{k|k-1}(\zeta )\right]⋃B_{k}$$

and the RFS of sensor measurements is:

(4) $$Z_{k}=\left[\underset{x\in X_{k}}{⋃}\Theta_{k}(x)\right]⋃C_{k}$$

where $\underset{\zeta \in X_{k-1}}{⋃}S_{k|k-1}(\zeta )$ is the survival target RFS inherited from the RFS $X_{k-1}$; $B_{k}$ is the birth target RFS; $\underset{x\in X_{k}}{⋃}\Theta_{k}(x)$ is the RFS of the detected target-originated measurements; and $C_{k}$ is the RFS of clutter.

### 2.2. The PHD Filter

In the PHD filter, the following common assumptions [6] are made:

*A.1:* each target state evolves and generates one measurement independently;

*A.2:* any clutter is Poisson distributed and independent of target-originated measurements;

*A.3:* the predicted multi-target RFS is Poisson distributed and independent.

Given the posterior intensity $D_{k-1}(x)$, the intensity function $D_{k|k-1}(x)$ is:

(5) $$D_{k|k-1}(x)=\gamma_{k|k-1}(x)+\int p_{s,k}(\tau )f_{k|k-1}(x|\tau )D_{k-1}(\tau )d\tau$$

and the posterior intensity $D_{k}(x)$ is:

(6) $$D_{k}(x)=(1-p_{D,k}(x))D_{k|k-1}(x)+\;\sum_{z\in Z_{k}}\frac{p_{D,k}(x)g_{k}\left(z|x\right)D_{k|k-1}(x)}{\kappa_{k}(z)+\int p_{D,k}(\tau )g_{k}\left(z|\tau \right)D_{k|k-1}(\tau )d\tau }$$

where $p_{D,k}(x)$ is the detection probability of an individual target with state x; $\gamma_{k|k-1}(\cdot )$ is the birth target PHD at time k; $\kappa_{k}(\cdot )$ is the intensity of the clutter RFS.

**Lemma** **1.** *If the multi-target PHD at time* k−1 *is represented as the sum of multiple PHDs with each PHD having the following Gaussian mixture form [6]:*

(7) $$D_{k-1}(x)=\sum_{i=1}^{N_{k-1}}W_{k-1}^{\left(i\right)}(x)$$

*with*

(8) $$W_{k-1}^{\left(i\right)}(x)=\sum_{j=1}^{N_{k-1}^{i}}\omega_{k-1}^{\left(i,j\right)}N\left(x;m_{k-1}^{\left(i,j\right)},P_{k-1}^{\left(i,j\right)}\right)$$

*then the predicted PHD will be*

(9) $$D_{k|k-1}(x)=\sum_{i=1}^{N_{k-1}}W_{k|k-1}^{\left(i\right)}(x)$$

*with*

(10) $$W_{k|k-1}^{\left(i\right)}(x)=\sum_{j=1}^{N_{k-1}^{i}}\omega_{k|k-1}^{\left(i,j\right)}N\left(x;m_{k|k-1}^{\left(i,j\right)},P_{k|k-1}^{\left(i,j\right)}\right)$$

*where*

(11) $$\omega_{k|k-1}^{\left(i,j\right)}=p_{S,k}\omega_{k-1}^{\left(i,j\right)}$$

*and* $m_{k|k-1}^{\left(i,j\right)}$ *is the predicted mean;* $P_{k|k-1}^{\left(i,j\right)}$ *is the predicted covariance*.

**Lemma** **2.** *Given the predicted PHD* $D_{k|k-1}(x)$ *and the measurements* $Z_{k}$*, the updated PHD [6] is:*

(12) $$D_{k}(x)=\sum_{i=1}^{N_{k-1}}W_{k}^{\left(i\right)}(x)$$

*with*

(13) $$W_{k}^{\left(i\right)}(x)=\sum_{j=1}^{N_{k-1}^{i}}\left(1-p_{D,k}\right)\omega_{k|k-1}^{\left(i,j\right)}N\left(x;m_{k|k-1}^{\left(i,j\right)},P_{k|k-1}^{\left(i,j\right)}\right)+\sum_{j=1}^{N_{k-1}^{i}}\sum_{z\in Z_{k}}\omega_{k}^{\left(i,j\right)}(z)N\left(x;m_{k}^{\left(i,j\right)}(z),P_{k}^{\left(i,j\right)}\right)$$

*where*

(14) $$\omega_{k}^{\left(i,j\right)}(z)=\frac{\omega_{k|k-1}^{\left(i,j\right)}p_{D,k}}{c(z)+\sum_{i=1}^{N_{k-1}}\sum_{j=1}^{N_{k-1}^{i}}\omega_{k|k-1}^{\left(i,j\right)}p_{D,k}N\left(z;h(m_{k|k-1}^{\left(i,j\right)}),S_{k|k-1}^{\left(i,j\right)}\right)}N\left(z;h(m_{k|k-1}^{\left(i,j\right)}),S_{k|k-1}^{\left(i,j\right)}\right)$$

**Remark** **1.** *As shown in the PHD recursions specified by Lemmas 1–2, the output from the PHD filter provides a multimodal density from which we need to estimate the states of the targets at each time, while it does not output target tracks. Moreover, the persistent target PHD and birth target PHD are jointly processed indifferently. In fact, the birth PHD reflects the nature of the birth target and is used for capturing newborn targets while the persistent PHD is updated for track maintenance of the surviving targets. Utilizing their difference is expected to further improve both tracking performance and calculation efficiency. Furthermore, all measurements (most of them are just clutter, for example in sea surveillance and ground tracking) are utilized to update the PHD, and hence most computational resources are wasted. These facts motivated us to establish the adaptive multi-PHD filter framework with track extraction and adaptive processing via both adaptive measurement partition and multi-PHD collaboration*.

## 3. Adaptive Collaborative GMPHD (ACo-GMPHD) Filter

**Figure 1:**
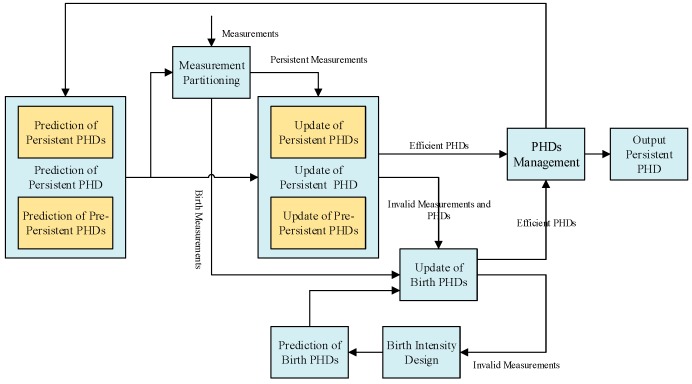
The adaptive collaborative Gaussian Mixture Probability Hypothesis Density (ACo-GMPHD) filter framework.

Figure 1 is the ACo-GMPHD framework consists of persistent PHDs, birth PHDs, and pre-persistent PHDs. Here, each PHD is specialized for a certain permanent/new-born/temporary target. In order to improve the computational efficiency, after the prediction of persistent PHDs and pre-persistent PHDs, the measurements in the surveillance area are adaptively partitioned into the persistent measurements and the birth measurements. Furthermore, the persistent PHDs and the pre-persistent PHDs are updated only using the persistent measurements, hence avoiding unnecessary data processing.

**Remark** **2.** *One may wonder whether the measurement partition in the ACo-GMPHD filter introduces several decision risks. In fact, the measurements away from the confidence region have little contribution to the persistent targets, and hence the weight of the Gaussian components would not be underestimated in updating the persistent PHD. In other words, the partition of the measurements has nothing to do with updating the persistent targets in setting the large-confidence region. For the birth target, when the confidence region is large, the birth measurements may be possibly partitioned into the persistent measurements, which may lead to missing the birth target. Here the measurement which does not contribute to the update of the persistent PHDs will be introduced into the birth measurement set, which can avoid the birth target missing. In general, adaptive measurement partition is effective as shown in the later simulation*.

### 3.1. Prediction of Persistent PHD

Given the persistent PHD set ${\left\{{\hat{W}}_{p,k-1}^{\left(i\right)},{\hat{l}}_{p,k-1}^{\left(i\right)},{\hat{\Xi }}_{p,k-1}^{\left(i\right)}\right\}}_{i=1}^{N_{p,k-1}}$ and the pre-persistent PHD set ${\left\{{\hat{W}}_{b/p,k-1}^{\left(i\right)},{\hat{l}}_{b/p,k-1}^{\left(i\right)},{\hat{\Xi }}_{b/p,k-1}^{\left(i\right)}\right\}}_{i=1}^{N_{b/p,k-1}}$, the target intensity is represented in the Gaussian mixture form of multiple PHDs:

(15) $$D_{k-1}^{p}(x)=\sum_{i=1}^{N_{p,k-1}}{\hat{W}}_{p,k-1}^{\left(i\right)}(x)+\sum_{i=1}^{N_{b/p,k-1}}{\hat{W}}_{b/p,k-1}^{\left(i\right)}(x)$$

with

(16) $${\hat{W}}_{p,k-1}^{\left(i\right)}(x)=\sum_{j=1}^{N_{p,k-1}^{i}}{\hat{\omega }}_{p,k-1}^{\left(i,j\right)}N\left(x;{\hat{m}}_{p,k-1}^{\left(i,j\right)},{\hat{P}}_{p,k-1}^{\left(i,j\right)}\right)$$

(17) $${\hat{W}}_{b/p,k-1}^{\left(i\right)}(x)=\sum_{j=1}^{N_{b/p,k-1}^{i}}{\hat{\omega }}_{b/p,k-1}^{\left(i,j\right)}N\left(x;{\hat{m}}_{b/p,k-1}^{\left(i,j\right)},{\hat{P}}_{b/p,k-1}^{\left(i,j\right)}\right)$$

where ${\hat{W}}_{p,k-1}^{\left(i\right)}(x)$ and ${\hat{W}}_{b/p,k-1}^{\left(i\right)}(x)$ denote the *i*-th persistent PHD and the *i*-th pre-persistent PHD; ${\hat{l}}_{p,k-1}^{\left(i\right)}$ and ${\hat{l}}_{b/p,k-1}^{\left(i\right)}$ are the corresponding labels; and ${\hat{\Xi }}_{p,k-1}^{\left(i\right)}$ and ${\hat{\Xi }}_{b/p,k-1}^{\left(i\right)}$ are the corresponding deleting thresholds, respectively.

According to Lemma 1, the predicted persistent PHD is:

(18) $$D_{k|k-1}^{p}(x)=\sum_{i=1}^{N_{p,k-1}}W_{p,k|k-1}^{\left(i\right)}(x)+\sum_{i=1}^{N_{b/p,k-1}}W_{b/p,k|k-1}^{\left(i\right)}(x)$$

where

(19) $$W_{p,k|k-1}^{\left(i\right)}(x)=\sum_{j=1}^{N_{p,k-1}^{i}}\omega_{p,k|k-1}^{\left(i,j\right)}N\left(x;m_{p,k|k-1}^{\left(i,j\right)},P_{p,k|k-1}^{\left(i,j\right)}\right)$$

(20) $$W_{b/p,k|k-1}^{\left(i\right)}(x)=\sum_{j=1}^{N_{p,k-1}^{i}}\omega_{b/p,k|k-1}^{\left(i,j\right)}N\left(x;m_{b/p,k|k-1}^{\left(i,j\right)},P_{b/p,k|k-1}^{\left(i,j\right)}\right)$$

### 3.2. Prediction of Birth PHDs

According to reference [19], we choose the scheme of calculating birth PHD $D_{k-1}^{b}$:

(21) $$D_{k-1}^{b}=\sum_{i=1}^{N_{b,k-1}}{\hat{W}}_{b,k-1}^{\left(i\right)}(x)$$

with

(22) $${\hat{W}}_{b,k-1}^{\left(i\right)}(x)=\sum_{j=1}^{N_{b,k-1}^{i}}{\hat{\omega }}_{b,k-1}^{\left(i,j\right)}N\left(x;{\hat{m}}_{b,k-1}^{\left(i,j\right)},{\hat{P}}_{b,k-1}^{\left(i,j\right)}\right)$$

where ${\hat{\omega }}_{b,k-1}^{\left(i,j\right)}$, ${\hat{m}}_{b,k-1}^{\left(i,j\right)}$, and ${\hat{P}}_{b,k-1}^{\left(i,j\right)}$ are calculated based on the measurements which were not utilized for track update at time k−1.

According to Lemma 1, the predicted birth PHD is:

(23) $$D_{k|k-1}^{b}=\sum_{i=1}^{N_{b,k-1}}W_{b,k|k-1}^{\left(i\right)}(x)$$

where

(24) $$W_{b,k|k-1}^{\left(i\right)}(x)=\sum_{j=1}^{N_{p,k-1}^{i}}\omega_{b,k|k-1}^{\left(i,j\right)}N\left(x;m_{b,k|k-1}^{\left(i,j\right)},P_{b,k|k-1}^{\left(i,j\right)}\right)$$

### 3.3. Measurement Partition

As the integral of the PHD equals the expectation of the target number in the surveillance region, the PHD should be updated by using the target-oriented measurements instead of all the measurements. Different kinds of PHDs such as the persistent PHD and the birth PHD should be updated based on the different measurement sets. Thus, we partitioned the measurement space through utilizing the information of the Gaussian terms.

The innovation covariances of the persistent and pre-persistent PHDs are:

(25) $$S_{p,k|k-1}^{\left(i,j\right)}=H_{k}P_{p,k|k-1}^{\left(i,j\right)}H_{k}^{T}+R_{k},s=1,\ldots ,N_{p,k-1},t=1,\ldots ,N_{p,k-1}^{s}$$

(26) $$S_{b/p,k|k-1}^{\left(s,t\right)}=H_{k}P_{b/p,k|k-1}^{\left(s,t\right)}H_{k}^{T}+R_{k},s=1,\ldots ,N_{b,k-1},t=1,\ldots ,N_{b,k-1}^{s}$$

The shortest Mahalanobis distance between a real measurement $z_{k}^{i}\in Z_{k}$ and the predicted measurement is:

(27) dk=min(mini=1,…,Np,k−1j=1,…,Np,k−1i(zki−Hkmp,k|k−1(i,j))T(Sp,k|k−1(i,j))−1(zki−Hkmp,k|k−1(i,j)),dk=minmins=1,…,Nb/p,k−1t=1,…,Nb/p,k−1s(zki−Hkmb/p,k|k−1(s,t))T(Sb/p,k|k−1(s,t))−1(zki−Hkmb/p,k|k−1(s,t)))

If $d_{k}<\tau^{\alpha }$, then the measurement $z_{k}^{i}$ will be assigned to the persistent measurement set $Z_{k}^{p}\subseteq Z_{k}$, where the threshold $\tau^{\alpha }$ denotes the α quantile of the upper-tail of a chi-squared distribution with $n_{z}$ degrees of freedom [1], where $n_{z}$ is the measurement dimension.

The birth measurement set $Z_{k}^{b}\subseteq Z_{k}$ is

(28) $$Z_{k}^{b}=\left\{z_{k}^{i}\in Z_{k}|z_{k}^{i}\notin Z_{k}^{p}\right\}$$

### 3.4. Update of Persistent PHD

According to Lemma 2, the persistent PHD is updated based on the persistent measurement set $Z_{k}^{p}$:

(29) $$D_{k|k}^{p}(x)=\sum_{i=1}^{N_{p,k-1}}W_{p,k|k}^{\left(i\right)}(x)+\sum_{i=1}^{N_{b/p,k-1}}W_{b/p,k|k}^{\left(i\right)}(x)$$

with

(30) $$W_{p,k|k}^{\left(i\right)}(x)=\left(1-p_{D,k}+\sum_{z\in Z_{k}^{p}}\frac{p_{D,k}g_{k}(z|x)}{L_{p,k}(z)}\right)W_{p,k|k-1}^{\left(i\right)}(x)$$

(31) $$W_{b/p,k|k}^{\left(i\right)}(x)=\left(1-p_{D,k}+\sum_{z\in Z_{k}^{p}}\frac{p_{D,k}g_{k}(z|x)}{L_{p,k}(z)}\right)W_{b/p,k|k-1}^{\left(i\right)}(x)$$

(32) $$L_{p,k}(z)=c_{p}(z)+\int p_{D,k}g(z|x)D_{k|k-1}^{p}(x)dx$$

where $c_{p}(z)$ is the clutter intensity in the survival region; $l_{p.k}^{\left(i\right)}=l_{p.k-1}^{\left(i\right)}$; $\Xi_{p.k}^{\left(i\right)}=\Xi_{p.k-1}^{\left(i\right)}$; $l_{b/p.k}^{\left(i\right)}=l_{b/p.k-1}^{\left(i\right)}$; and $\Xi_{b/p.k}^{\left(i\right)}=\Xi_{b/p.k-1}^{\left(i\right)}$.

The corresponding measurement weight of the persistent measurement is:

(33) $$\Psi_{k|k}^{p}(z)=\frac{p_{D,k}g_{k}(z|x)D_{k|k-1}^{p}(x)}{L_{p,k}(z)}=\sum_{i=1}^{N_{p,k-1}}\sum_{j=1}^{N_{p,k-1}^{i}}\omega_{p,k|k}^{\left(i\right)}(z)+\sum_{i=1}^{N_{b/p,k-1}}\sum_{j=1}^{N_{b/p,k-1}^{i}}\omega_{b,k|k}^{\left(i\right)}(z)$$

The measurement set for updating the birth PHDs is defined as:

(34) $$Z_{k}^{p/b}=\left\{z\in Z_{k}^{p}|\Psi_{k|k}^{p}(z)<T_{z}\right\}$$

where $T_{z}$ is the threshold for selecting the invalid measurements. Now the birth measurement set contains two parts:

(35) $$Z_{k}^{b}=Z_{k}^{b}⋃Z_{k}^{p/b}$$

The integrals of the persistent and pre-persistent PHDs are:

(36) $${\hat{\omega }}_{p,k}^{\left(i\right)}=\sum_{j=1}^{N_{p,k-1}^{i}}(1-p_{D,k})\omega_{p,k|k-1}^{\left(i,j\right)}+\sum_{j=1}^{N_{p,k-1}^{i}}\sum_{z\in Z_{k}^{p}}\omega_{p,k|k}^{\left(i,j\right)}(z)$$

(37) $${\hat{\omega }}_{b/p,k}^{\left(i\right)}=\sum_{j=1}^{N_{b/p,k-1}^{i}}(1-p_{D,k})\omega_{b/p,k|k-1}^{\left(i,j\right)}+\sum_{j=1}^{N_{b/p,k-1}^{i}}\sum_{z\in Z_{k}^{p}}\omega_{b/p,k|k}^{\left(i,j\right)}(z)$$

Denote the persistent PHD index set by $I_{p}=\{1,\ldots ,N_{p,k-1}\}$ and the pre-persistent PHD index set by $I_{b/p}=\{1,\ldots ,N_{b/p,k-1}\}$. The invalid persistent and pre-persistent PHD index sets are:

(38) $$L_{p}=\left\{i\in I_{P}|{\hat{\omega }}_{p,k}^{\left(i\right)}<T_{0}\right\}$$

(39) $$L_{b/p}=\left\{i\in I_{b/P}|{\hat{\omega }}_{b/p,k}^{\left(i\right)}<T_{0}\right\}$$

where $T_{0}$ is the upper-bound threshold of the weight of the invalid PHD. The invalid PHDs are treated as the additional birth PHDs in the following processing.

### 3.5. Update of Birth PHDs

For any birth PHD, its prediction is:

(40) $$D_{k|k-1}^{b}(x)=\sum_{i=1}^{N_{b,k-1}}W_{b,k|k-1}^{\left(i\right)}(x)+\sum_{i\in L_{p}}W_{p,k|k-1}^{\left(i\right)}(x)+\sum_{i\in L_{b/p}}W_{b/p,k|k-1}^{\left(i\right)}(x)=\sum_{i=1}^{N_{b,k}}W_{b,k|k-1}^{\left(i\right)}(x)$$

with

$$N_{b,k}=N_{b,k-1}+\left|L_{p}\right|+\left|L_{b/p}\right|$$

where $\left|L_{p}\right|$ represents the cardinality of the persistent targets; $\left|L_{b/p}\right|$ represents the cardinality of the invalid persistent targets.

According to Lemma 2, the birth PHDs are updated using the birth measurements $Z_{k}^{b}$:

(41) $$D_{k|k}^{b}(x)=\sum_{i=1}^{N_{b,k}}W_{b,k|k}^{\left(i\right)}(x)$$

(42) $$W_{b,k|k}^{\left(i\right)}(x)=\left(1-p_{D,k}+\sum_{z\in Z_{k}^{b}}\frac{p_{D,k}g_{k}(z|x)}{c_{b}(z)+\int p_{D,k}g_{k}\left(z|x\right)D_{k|k-1}^{b}(x)dx}\right)W_{b,k|k-1}^{\left(i\right)}(x)$$

where $c_{b}(z)$ is the clutter intensity in the birth region.

Then the weight of the birth measurement and the integral of the birth PHD are calculated as:

(43) $$\Psi_{k|k}^{b}(z)=\frac{p_{D,k}g_{k}(z|x)D_{k|k-1}^{b}(x)}{L_{b,k}(z)}$$

(44) $${\hat{\omega }}_{b,k}^{\left(i\right)}=\sum_{j=1}^{N_{b,k-1}^{i}}\left(1-p_{D,k}\right)\omega_{b,k|k-1}^{\left(i,j\right)}+\sum_{j=1}^{N_{b,k-1}^{i}}\sum_{z\in Z_{k}^{p}}\omega_{b,k|k}^{\left(i,j\right)}(z)$$

and the invalid measurement set is constructed:

(45) $$Z_{k,BI}={\left\{z\in Z_{k}^{b}|\Psi_{k|k}^{b}(z)<T\right\}}_{z}$$

Denote $J_{b,k}=\left|Z_{k,BI}\right|$ as the number of birth PHDs at the next time step.

### 3.6. PHDs Management

#### 3.6.1. Birth PHDs Management

If a birth PHD $W_{b,k|k}^{\left(i\right)}(x)$ has a large enough weight, such as ${\hat{\omega }}_{b,k}^{\left(i\right)}\geq T_{0}$, it can be reclassified as a pre-persistent PHD. Then, the pre-persistent PHD set $\left\{W_{b/p,k|k}^{\left(j\right)}(x),j=1,\ldots ,N_{k-1}^{b/p}\right\}$ is augmented by $W_{b,k|k}^{\left(i\right)}(x)$ in the birth PHD set $\left\{W_{b,k|k}^{\left(i\right)}(x),i=1,\ldots ,J_{b,k-1}\right\}$.

#### 3.6.2. Pre-Persistent PHDs Management

If a pre-persistent PHD $W_{b/p,k|k}^{\left(j\right)}(x)$ has a large enough weight, such as ${\hat{\omega }}_{b/p,k}^{\left(j\right)}\geq T_{0}$, it can be reclassified as a persistent PHD. Then, the persistent PHD set $\left\{W_{p,k|k}^{\left(j\right)}(x),j=1,\ldots ,N_{k-1}^{p}\right\}$ is augmented by $W_{b/p,k|k}^{\left(j\right)}(x)$ in the pre-persistent PHD set $\left\{W_{b/p,k|k}^{\left(j\right)}(x),j=1,\ldots ,N_{k-1}^{b/p}\right\}$.

#### 3.6.3. Persistent PHDs Management

A persistent PHD with a large enough large weight, for example $\omega_{p,k}^{\left(i\right)}\geq T_{0}$, is outputted as the stable track. Otherwise, if $\omega_{p,k}^{\left(i\right)}<T_{0}$ up to successive $E_{0}$ time instants, then such a persistent PHD is considered as the terminated track.

### 3.7. Birth Intensity Design

Here we adopt the strategy previously described in reference [20] as follows. A one-step initialization method is utilized to select the reliable birth intensity components for the next time step. The measurements near the current multi-target states are deleted to reduce the unnecessary birth intensity components. Without velocity information, the a priori velocity is zero-mean and has the covariance determined based on the maximum expected velocity (For more details, see reference [21]). The invalid measurement set $Z_{k,BI}$ is assigned to determine the birth PHD at time k:

(46) $$D_{k}^{b}(x)=\sum_{i=1}^{J_{b,k}}{\hat{W}}_{b,k}^{\left(i\right)}(x)$$

with

(47) $${\hat{W}}_{b,k}^{\left(i\right)}(x)={\hat{\omega }}_{b,k}^{\left(i\right)}N\left(x;{\hat{m}}_{b,k}^{\left(i\right)},{\hat{P}}_{b,k}^{\left(i\right)}\right)$$

(48) $${\hat{m}}_{b,k}^{\left(i\right)}={\left[z_{i},0\right]}^{T}\text{ for }z_{i}\in Z_{k,BI}$$

(49) $${\hat{P}}_{b,k}^{\left(i\right)}=\left[\begin{array}{cc} R_{k} & 0\\ 0 & V_{\max}^{2}I_{M}/3 \end{array}\right]$$

where the Gaussian term weight ${\hat{\omega }}_{b,k}^{i}$ is calculated according to reference [20]; $I_{M}$ is an *M*-by-*M* identity matrix; and M is the cardinality of the measurement set $Z_{k,BI}$.

### 3.8. Output Persistent PHD

At different times, the Gaussian terms with the same label represent the same target. The output track information at time k is the state ${\hat{m}}_{p,k}^{\left(i\right)}$ and covariance ${\hat{P}}_{p,k}^{\left(i\right)}$ of the persistent PHDs respectively:

(50) $${\hat{m}}_{p,k}^{\left(i\right)}=\frac{\int xW_{p,k|k}^{\left(i\right)}(x)dx}{\int W_{p,k|k}^{\left(i\right)}(x)dx}\;=\sum_{j=1}^{N_{p,k-1}^{i}}\frac{(1-p_{D,k})\omega_{p,k|k-1}^{\left(i,j\right)}}{{\hat{\omega }}_{p,k}^{\left(i\right)}}m_{p,k|k-1}^{\left(i,j\right)}+\sum_{j=1}^{N_{p,k-1}^{i}}\sum_{z\in Z_{k}^{p}}\frac{\omega_{p,k|k}^{\left(i,j\right)}(z)}{{\hat{\omega }}_{p,k}^{\left(i\right)}}m_{p,k|k}^{\left(i,j\right)}(z)$$

(51) $$\begin{array}{ll} {\hat{P}}_{p,k}^{\left(i\right)} & =\frac{\int (x-{\hat{m}}_{p,k}^{\left(i\right)}){\left(x-{\hat{m}}_{p,k}^{\left(i\right)}\right)}^{T}W_{p,k|k}^{\left(i\right)}(x)dx}{\int W_{p,k|k}^{\left(i\right)}(x)dx}\\ & =\sum_{j=1}^{N_{p,k-1}^{i}}\frac{(1-p_{D,k})\omega_{p,k|k-1}^{\left(i,j\right)}}{{\hat{\omega }}_{p,k}^{\left(i\right)}}\left[P_{p,k|k-1}^{\left(i,j\right)}+(m_{p,k|k-1}^{\left(i,j\right)}-{\hat{m}}_{p,k}^{\left(i\right)}){\left(m_{p,k|k-1}^{\left(i,j\right)}-{\hat{m}}_{p,k}^{\left(i\right)}\right)}^{T}\right]\\ & +\sum_{j=1}^{N_{p,k-1}^{i}}\sum_{z\in Z_{k}^{p}}\frac{\omega_{p,k|k}^{\left(i,j\right)}(z)}{{\hat{\omega }}_{p,k}^{\left(i\right)}}\left[P_{p,k|k}^{\left(i,j\right)}+(m_{p,k|k}^{\left(i,j\right)}(z)-{\hat{m}}_{p,k}^{\left(i\right)}){\left(m_{p,k|k}^{\left(i,j\right)}(z)-{\hat{m}}_{p,k}^{\left(i\right)}\right)}^{T}\right] \end{array}$$

**Remark** **3.** *The analysis for the complexity of the ACo-GMPHD is an open issue due to the measurement partition is random. In principle, the ACo-GMPHD and the standard GM-PHD have the similar process flowchart and hence their calculation burden is similar. Differing from the standard GM-PHD, the ACo-GMPHD partitions all measurements into smaller sets and separately processes them. Since the computational complexity of the standard GM-PHD increases exponentially with respect to the number of the related measurements, the ACo-GMPHD is expected to be more cost-efficient, which coincides with the simulation result*.

## 4. Simulation Analysis

**Figure 2:**
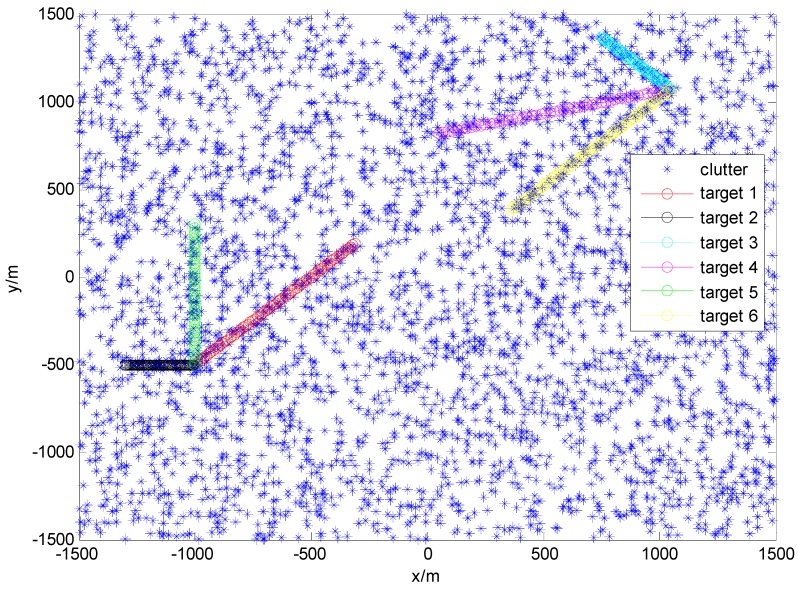
Trajectories of the true targets and the clutters.

To verify the systematic performance of the ACo-GMPHD filter, we compared it with the standard GMPHD filter which utilizes a priori birth target intensity and the GMPHD-I filter [20] via an MTT simulation scenario, similar to reference [8]. The difference is that six targets are considered here, instead of the three targets in reference [8]. The initial states, appearance, and disappearance of each target are given in Table 1. True trajectories are shown in Figure 2.

The detailed scenario parameters are given in Table 2.

Priori birth target intensity $\gamma_{k|k-1}(x)=0.1N(x,x_{1,}P)+0.1N(x,x_{2,}P_{\gamma })$ is provided for the standard GMPHD filter with $x_{1}={\left[-1000\;m,-500\;m,0\;m/s,0\;m/s\right]}^{T}$, $x_{2}={\left[1050\;m,1070\;m,0\;m/s,0\;m/s\right]}^{T}$, and $P_{\gamma }=diag\left\{100,100,100,100\right\}$.

We used a performance evaluation metric called the optimal subpattern assignment (OSPA) distance, which is specialized for the MTT filter accuracy test [22]. We selected OSPA parameters: p=2 and c=100.

We ran 100 Monte Carlo (MC) trials for each filter to obtain the OSPA distance and the averaged computational cost, and obtained the track-valued estimates in one MC trial. In addition, we validated the performance of each filter in the case of different clutter densities.

**Figure 3:**
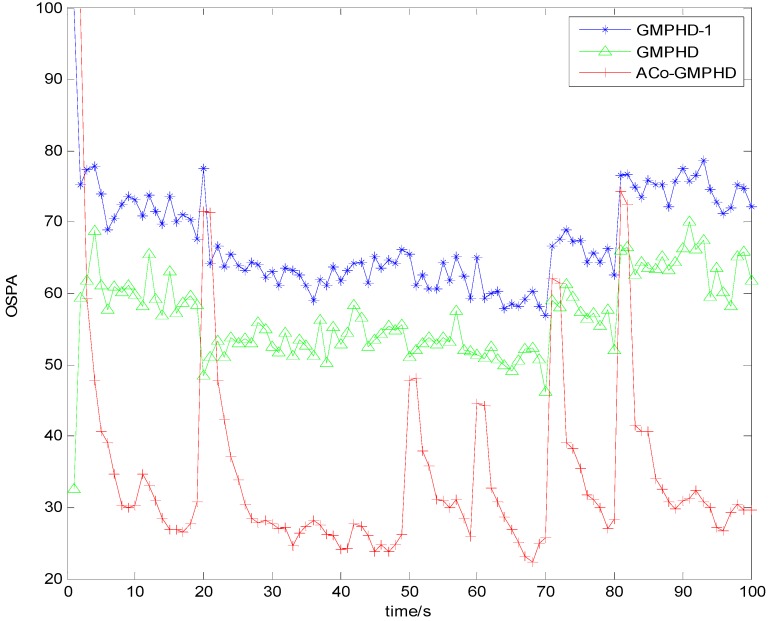
Monte Carlo average of the OSPA distance with a detection probability of 0.9.

**Figure 4:**
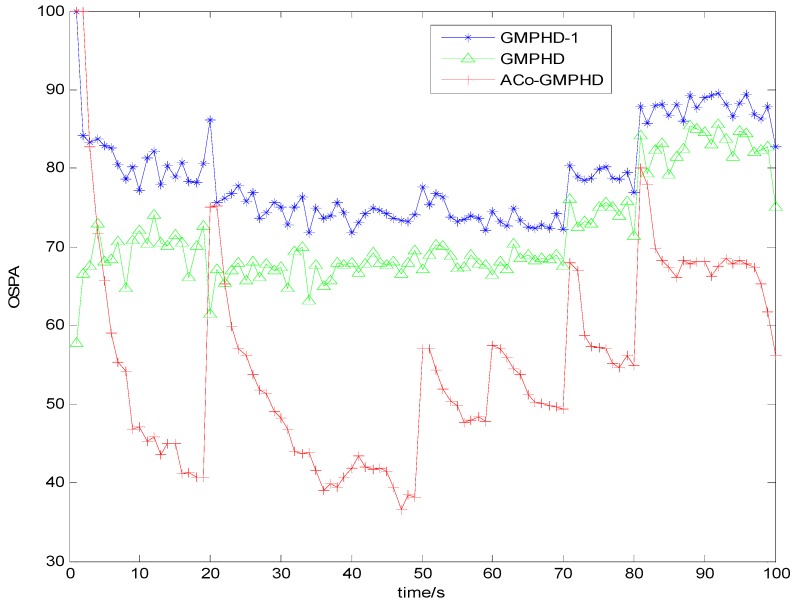
Monte Carlo average of the OSPA distance with a detection probability of 0.7.

Figure 3 and Figure 4 show the Monte Carlo average of the OSPA distance with detection probabilities of 0.9 and 0.7, respectively. Compared with the other two filters, the ACo-GMPHD almost always had the lowest OSPA distance, reflecting the effectiveness of the proposed adaptive multi-PHD collaboration and measurement partition.

**Figure 5:**
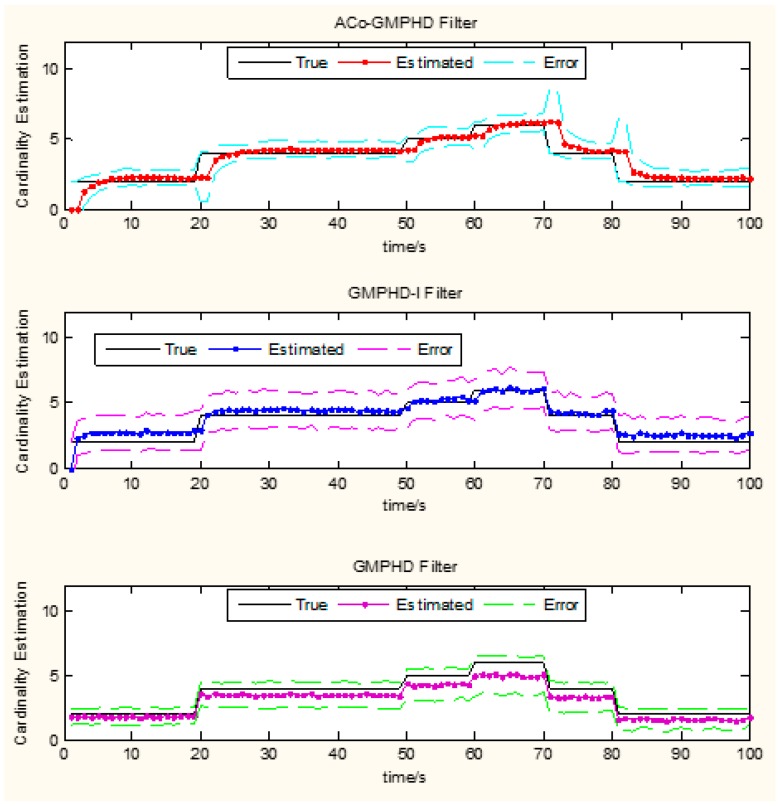
Monte Carlo average estimates of the number of targets. Estimated number (**solid lines**), standard deviation (**dashed lines**).

As shown in Figure 3 and Figure 4, there are five OSPA peaks in the ACo-GMPHD at times 20, 50, 60, 70, and 80 s, corresponding to the target appearances and disappearances, respectively. Some peaks are even higher than that of the standard GMPHD, and are always smaller than that of the GMPHD-I. The explanation for this is that:

- the ACo-GMPHD does not utilize a priori information of the birth target. At the moment that a target appears, the clutter and birth target measurements are hardly distinguishable without the support of the subsequent measurements. Thus, the birth target measurements may be treated as the clutter, and hence possibly leads to a delay in the cardinality estimation, as shown in Figure 5 when a target is newly born, which causes the peak of the OSPA distance. This is the cost of the measurement partition.
- the GMPHD-I also does not utilize a priori information of the birth target. However, due to the absence of adaptation and collaboration compared with the ACo-GMPHD, the GMPHD-I is the worst regarding the OSPA measure.

**Figure 6:**
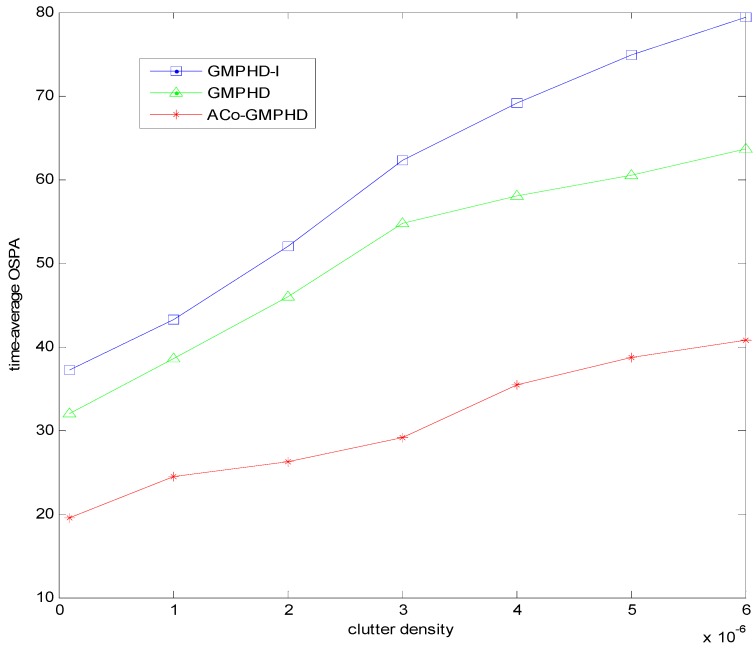
Time-averaged OSPA distance in one run of each filter for varying clutter densities.

Furthermore, we present the time-averaged OSPA versus the clutter density in Figure 6. With the increase of clutter density, the OSPA distance of the ACo-GMPHD filter gradually increases, but it is still lower than that of other two comparison algorithms.

**Figure 7:**
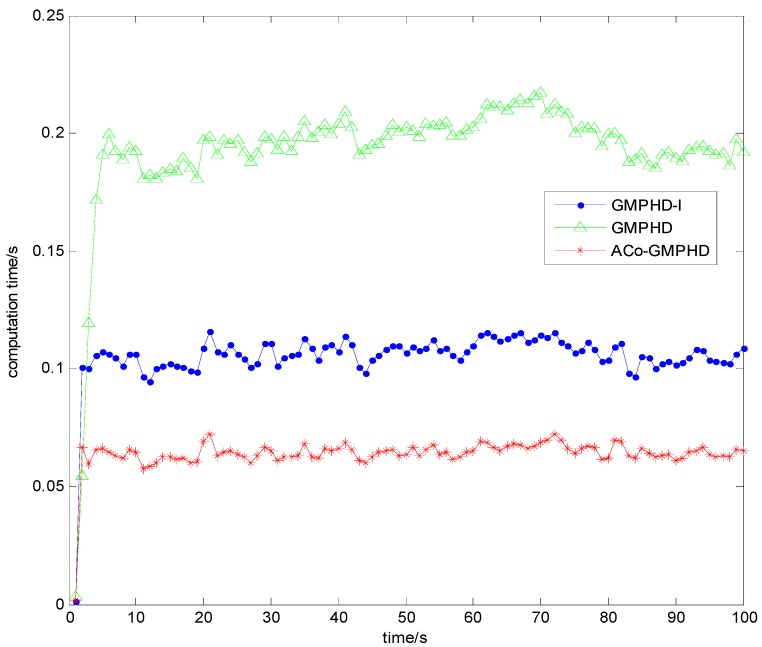
The computation time of the three filters.

For each time step, the averaged computation time (ACT) is shown in Figure 7. Obviously, the ACo-GMPHD filter significantly decreased the computational burden, compared with the standard PHD or GMPHD-I filters. The ACT of the ACo-GMPHD filter is about 0.65 s, much smaller than the measurement sampling period of 1 s.

**Figure 8:**
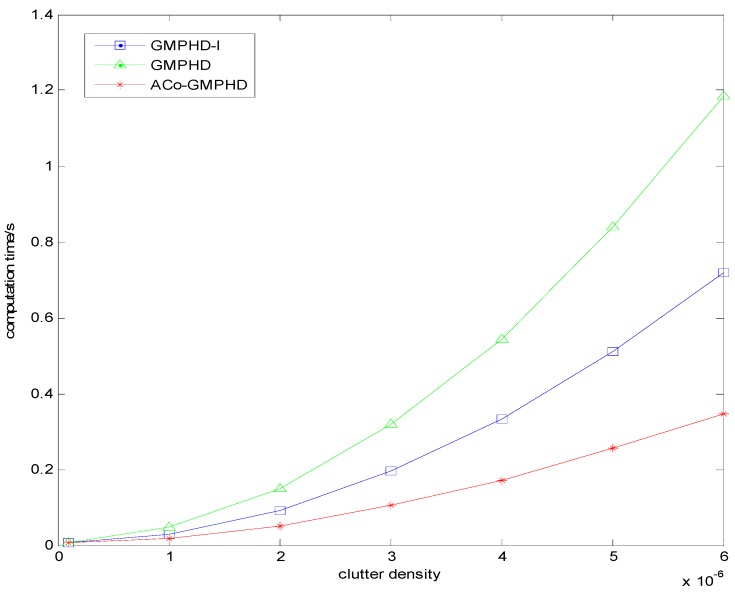
The time-averaged computation time under different clutter densities.

Figure 8 plots the curves of the ACT versus the clutter density. As the clutter density increases, the ACTs of all the filters increase; however, the ACo-GMPHD filter has the lowest rate of increase, implying that it is more suitable for the dense clutter case.

## 5. Conclusions

In this paper, we proposed the ACo-GMPHD filter with automatic track extraction, which was shown to be satisfactory for multi-target tracking. It costs less computational time and has better OSPA, except for some undesirable peaks at the moment of birth targets appear. Hence, one possible avenue for future research is to introduce priori information about birth targets, which would be helpful in order to better discern the target and the clutter, and hence more effectively reduce the corresponding OSPA peaks. Additionally, the threshold at the step of PHDs management was considered as constant in the proposed filter, and a possible future research study is to adaptively choose the threshold under some optimal performance index.

## Figures and Tables

**Table 1:** A List of Initial Target States.

Target Index	Appearing Time (s)	Disappearing Time (s)	Initial States (m, m, m/s, m/s)
1	1	70	(−1000, −500, 10, 10)
2	20	80	(−1000, −500, −5, 0)
3	20	80	(1050, 1070, −5, 5)
4	50	100	(1050, 1070, −20, −5)
5	60	100	(−1000, −500, 0, 20)
6	1	70	(1050, −1070, −10, −10)

**Table 2:** The Settings of the Simulation Scenario.

Category	Parameters	Value
Scenario	sampling period Δ	1 s
	region size (x-axis)	[−1500 km, 1500 km]
	region size (y-axis)	[−1500 km, 1500 km]
	clutter density λ	$4\times 10^{-6}m^{-2}$
	sensor noise covariance $R_{k}$	$\mathrm{diag}(100\;m^{2},100\;m^{2})$
	survival probability $p_{s,k}$	0.99
	detection probability $p_{D,k}$	0.9
ACo-GMPHD	state transition matrix *F*	$\left[\begin{array}{cc} I_{2} & \Delta I_{2}\\ 0_{2} & I_{2} \end{array}\right]$
	process noise standard deviation $\sigma_{v}$	5 m/s^2^
	process noise covariance $Q_{k}$	$\sigma_{v}^{2}\left[\begin{array}{cc} \frac{\Delta^{4}}{4}I_{2} & \frac{\Delta^{3}}{2}I_{2}\\ \frac{\Delta^{3}}{2}I_{2} & \Delta^{2}I_{2} \end{array}\right]$
	measurement matrix $H_{k}$	$\left[I_{2}0_{2}\right]$
	maximum target speed $v_{\max}$	50 m/s
	initial birth Gaussian weight ${\hat{\omega }}_{b,k}^{i}$	0.05
	weight threshold $T_{0}$	0.05
	measurement weight threshold $T_{z}$	0.1
	PHD deleting threshold $E_{0}$	2
